# Use of Electronic Cigarettes in Smoke-Free Spaces by Smokers: Results from the 2014–2015 Population Assessment on Tobacco and Health Study

**DOI:** 10.3390/ijerph17030978

**Published:** 2020-02-04

**Authors:** Zachary R. Dunbar, Gary Giovino, Binnian Wei, Richard J. O’Connor, Maciej L. Goniewicz, Mark J. Travers

**Affiliations:** 1 Roswell Park Comprehensive Cancer Center, Department of Health Behavior, Elm and Carlton Streets, Buffalo, NY 14263, USA; 2 Department of Community Health and Health Behavior, School of Public Health and Health Professions, University at Buffalo, The State University of New York, Buffalo, NY 14214-8028, USA; ggiovino@buffalo.edu

**Keywords:** electronic cigarettes, smoke-free locations, cessation, harm reduction

## Abstract

*Background*: Smoke-free air policies exist to protect users and nonusers from exposure to tobacco smoke. Although electronic nicotine delivery systems (ENDS) may expose passerby to nicotine and particulate matter, few US states regulate indoor use of ENDS. The purpose of this study was to investigate reported rationales for ENDS use and reported ENDS use in public smoke-free places by dual cigarette/ENDS users. *Methods*: A population of ENDS/cigarette co-users (*n* = 2051) was drawn from Wave 2 of the Population Assessment of Tobacco and Health (PATH) dataset (2014–2015). Harm reduction beliefs and cessation behavior of co-users were investigated as predictors of ENDS use in public smoke-free places using logistic regression. *Results:* Fifty-eight percent of dual users reported past 30-day ENDS use in public smoke-free places. Reported use of ENDS to cut down on cigarette smoking (OR: 2.38, 95% CI: 1.86, 3.05), as an alternative to quitting tobacco (OR: 1.71, 95% CI: 1.37, 2.13), or because of belief that ENDS help people to quit cigarettes (OR: 1.52, 95% CI: 1.20, 1.92) were significantly associated with increased odds of ENDS use in smoke-free places. *Conclusions:* Beliefs that ENDS were useful as cessation tools or posed modified risk to users and nonusers were associated with elevated odds of use ENDS in locations where conventional tobacco is prohibited. Due to limitations in the survey instrument, in-home ENDS use could not be directly assessed in this analysis. However, these self-reported findings suggest that use of ENDS in public places where cigarette use is prohibited is prevalent enough to be of concern for future regulation and enforcement efforts.

## 1. Introduction

Electronic nicotine delivery system (ENDS) use in smoke-free places has been previously assessed both as a primary motivator for ENDS use [1,2,3,4] as well as a reported condition under which ENDS are commonly used [5,6,7]. Viewing people using ENDS in a smoke-free place may provide a mechanism through which ENDS use can become normalized in smoke-free places [8]. ENDS use in public smoke-free places is of immediate concern because aerosol emitted from indoor ENDS use introduces nicotine, tobacco-specific nitrosamines (TSNAs), fine particles, and heavy metals into the immediate environment, and although there is a wide variety of ENDS which may emit disproportionate levels of these materials, their emission in ENDS aerosol may pose a risk of passive exposure to nearby nonusers [9,10,11,12,13,14]. Further, nicotine may also be deposited on surfaces in cases where ENDS use occurs indoors [15], potentially leading to passive exposure to nicotine and other ENDS aerosol constituents [16,17]. Passive exposure to nicotine may also be associated with developmental impairments in children, such as decreased motor function, [18] lower standardized test scores, [19] and higher risk of developing learning disabilities [20]. Prolonged TSNA exposure has been associated with increased risk of lung and esophageal cancer development in animal models [21,22] and is likely a contributing factor to the increased incidence of adenocarcinoma of the lung in adults over the past two decades [23]. Similarly, fine particles have been identified as a cause of lung inflammation and irritation when inhaled in large quantities [24]. Finally, the health effects of heavy metals introduced into the indoor environment by ENDS aerosol are widely unknown [25,26,27]. The emission of each of these chemical species by ENDS use in homes or enclosed public places is of particular concern, as infants and small children are the population most at-risk for exposure to each of the aforementioned pollutants [28,29]. These deleterious health outcomes highlight the need for greater research into the frequency and motivations for use of ENDS in public smoke-free places.

This analysis examines when and why ENDS are used in order to understand the factors that may drive ENDS use in public smoke-free places. While the quantity of scientific literature on determinants of electronic nicotine delivery system (ENDS) use has grown immensely over the past five years, [6,7,30,31] little is known about how potential motivators for ENDS use are related to use of these novel devices in smoke-free places. In general, ENDS use has been investigated as a potential cessation tool, with research suggesting that a primary reason for ENDS use among cigarette smokers may be to reduce or quit tobacco use [1,2,32,33]. Further, some research has shown that ENDS may be used to supplement or replace cigarettes in place of complete tobacco cessation [3,4,33,34]. Similarly, at least one study has theorized that clean indoor air laws may contribute to the selection of ENDS products whose use is harder to detect in locations with restrictions on vaping [6]. The belief that ENDS are more socially acceptable to use in public than conventional cigarettes [1,5] or the opinion that ENDS are less harmful than conventional cigarettes [2,3,4,32,33,35,36] have also been implicated as significant predictors of general ENDS use. Each of these determinants of ENDS use may influence ENDS device preference, as the availability of flavored e-products [1,35] and variety of ENDS designs [6] are each also popular motivations for ENDS use. Further, as explored in greater detail below, product characteristics may also drive safety and efficacy of a given product.

Although the FDA possesses regulatory authority over ENDS design and marketing, most clean air legislation and enforcement lies under the jurisdiction of state, regional, and local governments. In 2009, the Tobacco Control Act was passed, which gave the US Food and Drug Administration (FDA) regulatory authority over the marketing, distribution, and manufacturing of tobacco products available in the United States [37]. However, the Tobacco Control Act does not provide specific guidance on use of tobacco products in enclosed areas. Similarly, the ‘deeming rule’ published in August 2016 extends the regulatory authority of the FDA to include all e-cigarette devices [38]; however, the new deeming rule does not issue specific recommendations on the use of ENDS devices in indoor settings. Following the 2016 report of the Surgeon General, [29] the Centers for Disease Control and Prevention issued a call to action recommending the implementation of comprehensive clean indoor air laws that restrict the use of e-cigarettes in public, enclosed spaces; [39] however, introduction of state-level legislation regulating ENDS use in public smoke-free places has been sporadic. Comprehensive smoke-free indoor air laws (which restrict use of cigarettes in bars, restaurants, and workplaces) have been successfully implemented in twenty-five US states and territories (AZ, CA, DE, HI, IL, IA, KS, ME, MD, MA, MI, MN, MT, NE, NJ, NY, ND, OH, OR, RI, SD, UT, VT, WA, and WI, as well as Washington, DC, Puerto Rico, and the US Virgin Islands) [40]. However, despite these regulations, the use of ENDS in public smoke-free places continues to be widely unrestricted [41]. The purpose of this study was to investigate reported rationales for ENDS use and reported ENDS use in public smoke-free places by dual cigarette/ENDS users using cross-sectional data from a nationally representative cohort study in order to provide context for future tobacco control policy actions.

## 2. Materials and Methods

### 2.1. Sample

The Population Assessment of Tobacco and Health (PATH) Study was developed and implemented by the National Institutes of Health (NIH) and FDA and is a longitudinal cohort study of tobacco use and associated health outcomes [42]. There are 28,362 individuals in the PATH Wave 2 Adult (Age 18+) public-use data file, and this population is representative of the noninstitutionalized adult population of the United States [42]. Data for Wave 2 were collected between October 2014 and October 2015. Weighting was performed on the Wave 2 Adult dataset in order to adjust for differential probability of survey selection, nonresponse, and sampling frame bias [43]. Replicate weights were employed to correct for sampling frame bias and differential rates of nonresponse. Wave 2 weights correspond to weights applied during Wave 1 (implemented in 2013–2014), correcting for nonresponse and loss to follow up in the study cohort. The second wave of the PATH study was used in this assessment, as it was the most recent wave of the PATH study that was publicly available at study onset. All percentages given below are weighted according to the PATH guidelines unless otherwise noted.

### 2.2. Measures

Current established cigarette use in this study population was defined as individuals who have smoked 100 or more cigarettes in their lifetime and self-reported current ‘every day’ or ‘some days’ current cigarette use within the previous 30 days. Current ENDS users were defined as individuals who reported ever-use of ENDS and current use on ‘every day’ or ‘some days’ within the past 30 days. Daily ENDS users were defined as individuals who self-reported ‘every day’ ENDS use, and nondaily ENDS users were defined as participants who reported ‘some days’ ENDS use within past 30 days. The definitions for ‘some days’ and ‘every day’ cigarette and ENDS use given above match the definitions used in the derived variables in the PATH dataset [44]. Individuals who were both current daily or nondaily cigarette users and current daily or nondaily ENDS users were considered ‘dual’ users of cigarettes and ENDS. Statistics on use frequency is provided in Table 1 below. Participants who reported smoking only cigarettes or were sole users of ENDS products were omitted from this analysis. Frequency of ENDS and tobacco use was similarly deduced from the same self-report data used to populate the overall sample of dual ENDS/tobacco users. Of the 28,362 adults in the PATH Wave 2 dataset, 2051 individuals (7.23%) were defined as dual users. Use of ENDS in smoke-free places was defined as users who self-reported use of ENDS at times or in places (including homes and workplaces) when regular cigarettes could not be smoked at least one time over the past 30 days. This definition includes the use of ENDS in the users’ own homes. The PATH survey instrument asked this question to current ENDS users regardless of their frequency of use. Disposable ENDS users were defined as individuals who reported sole use of nonrechargeable ENDS devices. Cartridge ENDS users were defined as participants that responded that the device they owned or most frequently used contains e-liquid cartridges. Similarly, tank ENDS users were defined as individuals that reported owning or most frequently used e-devices with a tank design. Individuals were also asked about beliefs associated with general ENDS use, such as the belief that ENDS may help smokers to quit using tobacco. All other variables given in Table 1 below are presented in similar format and style as what was presented to PATH respondents during the interview.

### 2.3. Assessment

Statistical assessment was performed using SAS 9.4 (SAS Institute Incorporated, Cary, NC, USA). The distribution of sociodemographic characteristics as well as selected behavioral and attitudinal norms associated with ENDS use were assessed for current dual users, stratified by use of ENDS in smoke-free places. Attitudinal and behavioral characteristics associated with ENDS use were coded as dichotomous groups. Chi-square analyses were also performed in order to assess differences in reported use of ENDS in smoke-free places by each sociodemographic, attitudinal, or behavioral indicator variable. Fay’s method (using a coefficient of 0.3) was used alongside the weight replicates given in the PATH Wave 2 dataset in order to estimate the standard error of the weighting measure for each variable. Multivariable logistic regression was performed using PROC SURVEYLOGISTIC in order to assess the influence exerted by each sociodemographic, behavioral, or attitudinal predictor on odds of ENDS use in smoke-free places. Weighting factors and survey logistic procedures were utilized in accordance to the PATH Wave 2 user guidelines. Users who reported no ENDS use in smoke-free places formed the reference group for the logistic analysis. Crude and adjusted-odds ratios as well as 95% confidence intervals are given in the tables below for each predictor variable. Adjusted odds ratios correspond to beta estimates given from a multivariate logistic model containing the listed variable, with age, gender, race, ethnicity, education, and minutes to first cigarette included in the model as covariates; these data are summarized below in Table 2. Minutes to first cigarette was selected as an adjustment variable in this analysis due to its status as a proxy to indicate tobacco dependence; [45] further, minutes to first cigarette was given preference over analyst -ecoded variables such as minutes to first tobacco due to the large amount of missing data in the minutes to first ENDS variable. Minutes to first cigarette was coded as an ordinal variable, following group assignments: 0–5, 5–30, 31–60, and 61+ min. Variation inflation factors (VIF) were below 10 for each variable given in Table 2 below. All data analysis was performed using SAS 9.4 (SAS Institute Incorporated, Cary, NC, USA).

## 3. Results

The sociodemographic characteristics of the study population are given in Table 1. Of the 2051 dual users in the PATH Study, 1189 (58.0%) users reported using ENDS in smoke-free places at least one time over the past 30 days. A majority of the population was male (*n* = 1035, 53.4%), Caucasian (*n* = 1644, 81.92%), heterosexual (*n* = 1823, 89.7%), and had completed at least some college (n=1019, 50.3%) Most of the study population consisted of nondaily ENDS users (*n* = 1670, 80.9%) and daily cigarette smokers (n=1500, 72.9%), respectively. A majority (*n* = 1621, 79.8%) of the study population agreed that they used ENDS because of a belief that ENDS products may pose less risk to nearby nonusers compared to cigarettes, compared to 424 participants (19.9%) that did not. More study participants used cartridge or disposable ENDS designs (*n* = 1171, 58.1%) than tank systems and also agreed that they used ENDS because a variety of flavored products were available (*n* = 1425, 67.7%). Agreement with prompts of ENDS use due to perceived lack of smell (*n* = 1439, 71.4%), as a means to help quit smoking (*n* = 1423, 69.9%), an alternative to quitting tobacco altogether (*n* = 1132, 56.1%), and as a way to cut down on cigarette smoking (*n* = 1416, 70.1%) were also prevalent.

The logistic regression model output is provided in Table 2. Gender and education were both significantly associated with increased odds of past 30-day ENDS use in smoke-free places. Men were significantly less likely than women to report using ENDS in smoke-free places, adjusting for all covariates (OR: 0.76, 95% CI: 0.62, 0.93). Individuals whose highest level of education was a GED were significantly more likely to report ENDS use in smoke-free places (OR: 1.76, 95% CI: 1.09, 2.85) than participants who reported less than high school diploma educational status, adjusting for all covariates. Similarly, individuals who reported any level of college completion were significantly more likely to report ENDS use in smoke-free places (OR: 1.38 95% CI: 1.02, 1.88) compared to respondents with less than high school diploma education status and adjusting for all covariates. Individual preference for tank ENDS designs was significantly associated with increased odds of ENDS use in smoke-free places compared to disposable or cartridge designs, after adjustment for all covariates (OR: 1.47, 95% CI: 1.17, 1.85). Interestingly, frequency of cigarette use was not associated with change in odds of ENDS use in smoke-free places (OR: 0.65, 95%CI: 0.43, 1.00)

Among consumer characteristics, individuals who reported ENDS use because ENDS is “less harmful to nonusers than smoking” (OR: 2.10, 95% CI: 1.59, 2.76), “ENDS do not smell” (OR: 1.86, 95% CI: 1.43, 2.41), “[ENDS] are available in desirable flavors” (OR: 2.04, 95% CI: 1.62, 2.55), “[ENDS] help smokers to quit cigarettes” (OR: 1.52, 95% CI: 1.20, 1.92), believe that ENDS are an alternative to quitting tobacco (OR: 1.71, 95% CI: 1.37, 2.13) or believe that ENDS help to cut down on cigarette smoking (OR: 2.38, 95% CI: 1.86, 3.05) each were associated with increased likelihood of reported ENDS use in smoke-free places compared to individuals who did not share those beliefs, adjusting for all covariates. Dual users who self-reported ENDS use as a way of cutting down on cigarette use or as an alternative to quitting tobacco completely were both significantly more likely to report use of ENDS in smoke-free places compared to users who did not agree with those motivations for ENDS use. Cessation of tobacco use for one day or longer four or more times over the past year due to attempting to quit was also significantly associated with increased odds of ENDS use in smoke-free places (OR: 1.54, 95% CI: 1.12, 2.13). Participants who were daily ENDS users were significantly more likely to report ENDS use in smoke-free places compared to nondaily ENDS users, adjusting for all covariates (OR: 2.04, 95% CI: 1.39, 3.01).

## 4. Discussion

Although in-home use of ENDS products could not be directly assessed in this analysis, several characteristics of dual users were found to be significant predictors of ENDS use in other smoke-free places. Gender and education were both associated with significantly higher odds of reported ENDS use in smoke-free places. Men were less likely than women to report using ENDS in smoke-free places in this analysis. This finding may reflect greater awareness of ENDS use restrictions among men as men are more frequent users of ENDS than women. Participants who completed a GED or any college were more likely to report ENDS use in smoke-free spaces than individuals who did not complete high school; as was discussed above for gender, this finding may be due to the elevated use of ENDS in these populations. Individuals who agreed with beliefs that ENDS use is less harmful to passersby and reported using ENDS because of a belief that ENDS do not smell were both more likely to report using ENDS in smoke-free places than dual users who did not report either belief. This association is important for future policy actions, as various studies have suggested that ENDS emit detectable levels of nicotine and other pollutants into the immediate indoor environment, [16,17] as discussed above. These quantifiable levels of nicotine in the indoor environment suggest that even though e-cigarettes may pose an attenuated risk of nicotine exposure compared to conventional tobacco cigarettes, nicotine-containing ENDS devices do introduce nicotine and other chemical constituents into indoor settings. Therefore, more extensive national counter-marketing and education campaigns should be undertaken in order to prevent the advancement of beliefs that ENDS confer no risk of passive exposure to nicotine and other chemicals in users and nonusers alike. Further, if there are no regulations on ENDS use in a given clean air setting, it is worth investigating why all ENDS users are not using their devices in clean air indoor environments. It is possible that the harm reductive beliefs described above are not as well-entrenched in the user base as the data might suggest. Despite this disparity, counter-marketing programs would likely benefit users and nonusers alike by attenuating the harm reduction beliefs that this analysis observed to be associated with increased odds of ENDS use in smoke-free places.

ENDS product design was found to be a significant predictor of past 30-day ENDS use in smoke-free places. ENDS users who preferred a tank design were observed to have higher odds of reporting ENDS use in smoke-free places compared to cartridge or disposable ENDS users, adjusting for all covariates. However, the relationship between ENDS product preference and ENDS use in smoke-free places is not well understood. Smoke-free legislation may also retroactively drive ENDS device preference in that some ENDS users may prefer e-products that are easier to conceal, further clouding the association between product selection and use of ENDS in smoke-free settings. For example, a dual user may be prompted to use a disposable or cartridge device, as these products may be more discrete than larger tank-based vaporizers. The observed trend between product preference and odds of ENDS use in smoke-free places corroborates the trend reported by Yingst et al. (2019), suggesting that a complex association may exist between product design and use of ENDS in smoke-free places [6]. Similarly, it is of note that the ENDS marketplace evolves rapidly, and replacement of tank systems by more popular JUUL products and other easily concealed ENDS products in recent years may influence current motivations for ENDS use in smoke-free places. The complex nature of this relation highlights the need for future research on the nature of the interaction between ENDS product design preference, attitudes associated with ENDS use, and reported vaping in smoke-free places.

Cessation beliefs were strongly associated with ENDS use in smoke-free places in this analysis. ENDS use as a smoking cessation tool is currently a topic of much debate in the field of tobacco control [46,47,48,49]. As described above, cessation perceptions and previous cessation attempts played a more prominent role than frequency of cigarette use in predicting ENDS use in smoke-free places. Further, there was no observed difference in odds of ENDS use in smoke-free places between frequencies of ENDS and conventional tobacco use. These significant associations suggest that holding beliefs that ENDS may serve as cessation aids may in turn contribute to increased use of e-products in places where conventional cigarettes are prohibited. Similarly, cessation beliefs were not mutually exclusive, meaning respondents may have had multiple rationales for continued ENDS use. Further, there was a clear positive trend in the relationship between past 12 month quit attempts lasting longer than one day and use of ENDS in smoke-free places. Dual users with the highest rate of quit attempts over the past year were significantly more likely to report ENDS use in smoke-free places than those who reported zero quit attempts, which provides more evidence in favor of a relationship between ENDS use as cessation devices and use of ENDS in smoke-free places. In general, holding pro-cessation beliefs towards ENDS products was associated with elevated odds of ENDS use in this analysis. Further, as described above, use of ENDS in enclosed spaces may result in deposition of nicotine onto surfaces in the indoor environment [15]. Nicotine present in the passive indoor environment is a source of nicotine exposure in adults, and third-hand smoke may potentially provide a mechanism by which ENDS use in indoor environments prolongs nicotine addiction in adults who wish to quit smoking. Much more research is required in order better describe this potential relationship. Data presented above therefore suggest that incorporating ENDS into existing smoke-free air legislation regulating public spaces may aid dual users attempting cessation in quitting tobacco and may also reduce the amount of passive nicotine that dual and nonusers may be exposed to in enclosed spaces.

There are several notable limitations in this study. One primary limitation is that as of this writing, there are no available state or county identifiers in the public Wave 2 PATH dataset, which precludes the investigation of differences between smoking patterns of users in smoke-free versus nonsmoke-free states or counties and the appraisal of compliance with smoke-free indoor air policies at a comprehensive level. The survey design also did not specify if the smoke-free place was a public or private place, which is an important point of clarity, as smoke-free indoor legislation does not cover privately-owned homes, meaning that ENDS use in these locations is regulated solely by the homeowner. Further, individuals who own their own homes may and should exert final authority over their behavior in their own home—therefore, policy recommendations drafted by findings of this analysis should only extend to shared public spaces. The cross-sectional nature of this study design is also a limitation, in that this analysis does not allow for changes in ENDS/cigarette dual use, cessation attempts, ENDS product preferences, or any of the other attitudinal or behavioral characteristics described above to be evaluated longitudinally. This study was performed as a cross-sectional rather than a longitudinal analysis because ENDS policy evolves slowly: The smoke-free air policy environment for 2013 (corresponding to Wave 1 of the PATH study) was remarkably similar to the national policy environment in 2014 [50]. Further, the American ENDS marketplace is constantly evolving; because this wave of the PATH survey was implemented between 2014 and 2015, investigation of the exponential increase in popularity and market share of newer ENDS products and designs such as JUUL (introduced and popularized in 2017) is not possible. Similarly, another limitation of this assessment is that the Wave 3 dataset has been available for several months as of this writing. Although the cross-sectional nature of this analysis precludes any evaluation of long-term shifts in product use or attitudinal changes in regard to public smoke-free indoor air policy compliance, the gradual nature of smoke-free air legislation implementation in the United States creates a legislative environment where policy differences between annual waves of Population Assessment on Tobacco or Health data are minimal. Therefore, this study provides a novel snapshot of ENDS use in public smoke-free places using a nationally-representative sample and in doing so provides important context and rationale for federal, state, and municipal smoke-free policy.

## 5. Conclusions

This study provides important context for ENDS use in public smoke-free places. Evidence from this study suggests that ENDS use as a cessation tool may place nonusers at greater possible risk for deleterious health effects if substitutive ENDS use occurs in a location that is currently regulated by clean air laws. Further, harm-reduction beliefs in ENDS users were associated with increased odds of ENDS use in public smoke-free places, suggesting that cessation and harm-reduction beliefs both play a role in determining an individual’s likelihood to circumvent or disregard smoke-free air laws and policies. These findings both imply that a concerted education and counter-marketing campaign, combined with inclusion of ENDS into existing smoke-free policies, may provide critical protection for both ENDS/cigarette users and nonusers in public smoke-free places.

## Figures and Tables

**Table 1:** Distribution of sociodemographic and behavioral characteristics among dual users.

Variable	*n* (*N* = 2051)	Weighted Percent	Standard Error	*X^2^*	*p-Value*
Age					
18–24	539	17.97	0.84	18.59	<0.001
25–34	494	26.74	1.00		
35–44	399	22.03	1.12		
45–55	325	16.32	0.92		
55+	294	16.93	1.00		
Gender					
Male	1035	53.37	1.20	6.28	0.01
Female	1016	46.63	1.20		
Race					
Caucasian	1644	81.92	0.98	0.28	0.60
Black or Other	386	17.24	0.93		
Sexual Orientation					
LGBT+	208	9.15	0.69	6.73	0.08
Heterosexual	1823	89.74	0.77		
Marital Status					
Single	887	39.98	1.18	0.85	0.36
Married or other	1164	60.02	1.18		
Education Level					
Less than High School	300	13.39	0.74	19.18	<0.001
GED ^	243	11.67	0.82		
High School Diploma	474	23.95	1.22		
Any College	1019	50.28	1.53		
Yearly Income					
Missing	130	6.60	0.76	1.71	0.19
Below $25k	971	44.32	1.54		
Above $25K	950	49.09	1.65		
Residence Type					
Missing	541	18.12	0.86	0.98	0.61
Single family home	987	53.75	1.38		
Multi-family home or apartment	450	24.33	1.23		
Something else	73	3.80	0.55		
Own or Rent Home					
Own	677	35.28	1.33	6.71	0.15
Rent	1005	47.92	1.43		
Something else	351	15.87	0.91		
Home Smoking Policy					
Tobacco use prohibited at all times	1052	51.90	1.32	1.85	0.17
Any other policy	994	47.73	1.32		
ENDS Device Most Frequently Used					
Cartridge or disposable user	1171	58.14	1.41	25.48	<0.0001
Tank user	814	38.43	1.34		
Use ENDS Because: they might be less harmful to people around me than cigarettes					
Yes	1621	79.78	0.90	56.04	<0.0001
No	424	19.90	0.88		
Use/used e-cigarettes because: They don’t smell					
Yes	1439	71.38	1.04	43.80	<0.0001
No	607	28.37	1.04		
Use/used e-cigarettes because: It comes/came in flavors I like/liked					
Yes	1425	67.68	1.12	59.62	<0.0001
No	619	31.96	1.10		
Using them helps people to quit smoking cigarettes					
Yes	1423	69.85	1.25	26.93	<0.0001
No	624	29.95	1.24		
Use/used e-cigarettes as an alternative to quitting tobacco altogether					
Yes	1132	56.14	1.12	53.83	<0.0001
No	912	43.53	1.12		
Use/used e-cigarettes as a way of cutting down on cigarette smoking					
Yes	1416	70.09	1.07	115.65	<0.0001
No	633	29.85	1.06		
Count of times over past 12 months, stopped using tobacco for one day or longer because trying to quit					
0 times	1264	61.39	1.30	7.00	0.07
1 time	175	8.85	0.81		
2–3 times	390	19.06	0.95		
4+ times	222	10.70	0.73		
Minutes after waking to first cigarette					
less than 5 min	465	22.99	1.08	1.54	0.67
5–30 min	746	35.42	1.20		
31–60 min	385	19.26	1.00		
61+ min	443	21.69	1.21		
Minutes after waking to first puff on ENDS					
Missing	966	47.15	1.30	3.81	0.28
Less than 5 min	209	10.05	0.80		
5–30 min	270	13.24	0.87		
31–60 min	201	9.52	0.63		
61+ min	405	20.04	0.91		
Tobacco/ENDS Use					
Nondaily (Both)	353	17.43	0.97	83.25	<0.0001
Nondaily Cigarette, Daily ENDS	198	9.67	0.74		
Daily (Both)	183	9.46	0.66		
Daily Cigarette, Nondaily ENDS	1317	63.44	1.32		
Frequency of Daily ENDS Use (Regardless of Tobacco Use Frequency)					
Nondaily ENDS	1670	80.87	0.96	82.84	<0.0001
Daily ENDS	381	19.13	0.96		
Frequency of Daily Cigarette Use (Regardless of ENDS Use Frequency)					
Nondaily Cigarette User	551	27.1	1.24	5.19	0.02
Daily Cigarette User	1500	72.9	1.24		

* Missing values less than 5% of total population were excluded from Table 1 above. ^ General Education Diploma. In the United States, this corresponds to certification of academic skillsets equivalent to high school degree completion.

**Table 2:** Logistic model predicting odds of past 30-Day ENDS use in smoke-free locations—main effects.

Variable	Do Not Report Using ENDS in Smoke-Free Places			Report Using ENDS in Smoke-Free Places			Crude Model *			Adjusted Model **		
		*n* = 862			*n* = 1189							
*N* = 2051	*n*	%	SE	*n*	%	SE	Crude OR	95% CI LB	95% CI UB	Adj OR *	95% CI LB	95% CI UB
***Sociodemographic Characteristics***												
Age												
18–24	248	19.56	1.51	291	16.80	1.06	1	--	--	1	--	--
25–34	196	26.09	1.52	298	27.22	1.26	1.19	0.87	1.63	1.10	0.80	1.53
35–44	150	20.09	1.54	249	23.46	1.52	1.33	0.96	1.84	1.21	0.85	1.72
45–55	121	14.43	1.05	204	17.72	1.24	1.49	1.06	2.10	1.33	0.94	1.89
55+	147	19.82	1.61	147	14.79	1.23	0.77	0.55	1.10	0.70	0.49	1.00
Gender												
Male	463	56.87	1.82	572	50.79	1.68	0.75 *	0.61 *	0.91 *	0.76 *	0.62 *	0.93 *
Female	399	43.13	1.82	617	49.21	1.68	1	--	--	1	--	--
Race												
White alone	687	80.20	1.60	957	83.18	1.34	1	--	--	1	--	--
Black or other alone	167	18.97	1.60	219	15.96	1.29	0.79	0.57	1.09	0.77	0.55	1.07
Sexual Orientation												
LGBT +	75	7.48	0.97	133	10.38	0.95	1.49 *	1.01 *	2.20 *	1.37	0.93	2.02
Heterosexual	779	91.53	1.04	1044	88.42	1.05	1	--	--	1	--	--
Marital Status												
Single	383	41.22	1.88	504	39.07	1.63	1	--	--	1	--	--
Married or other	479	58.78	1.88	685	60.93	1.63	1.04	0.83	1.31	1.03	0.77	1.38
Education												
Less than High School	140	14.53	1.25	160	12.56	0.92	1	--	--	1	--	--
GED	90	9.79	1.11	153	13.06	1.13	1.83 *	1.16 *	2.90 *	1.76 *	1.09 *	2.85 *
High School Diploma	231	28.32	1.99	243	20.72	1.40	0.94	0.64	1.38	0.93	0.63	1.37
Any College	392	46.57	2.11	627	53.02	1.75	1.42 *	1.06 *	1.90 *	1.38 *	1.02 *	1.88 *
Yearly Income												
Below $25k	419	45.64	2.23	552	43.34	1.79	0.94	0.75	1.17	0.97	0.77	1.22
Above $25K	382	47.23	2.22	568	50.45	1.98	1	--	--	1	--	--
Residence Type												
Missing	250	19.90	1.58	291	16.80	1.06						
Single family home	392	51.18	1.96	595	55.65	1.77	1	--	--	1	--	--
Multi-family home or apartment complex	191	25.10	1.63	259	23.76	1.53	0.83	0.65	1.06	0.89	0.70	1.13
Something else	29	3.83	0.82	44	3.79	0.65	0.85	0.51	1.44	0.95	0.53	1.72
Own or Rent Home												
Own	302	37.36	2.18	375	33.75	1.50	1	--	--	1	--	--
Rent	413	46.42	2.13	592	49.03	1.60	1.17	0.93	1.47	1.25	0.96	1.62
None of the Above	136	14.88	1.33	215	16.60	1.19	0.29	0.93	1.80	1.39	0.97	2.00
Home Smoking Policy												
Tobacco use prohibited at all times	426	50.53	1.99	626	52.91	1.78	1	--	--	1	--	--
Any other policy	432	48.84	1.99	562	46.91	1.77	0.92	0.73	1.17	1.05	0.82	1.36
***Device Preference and ENDS Use Rationale***												
ENDS Device Most Frequently Used												
Cartridge or disposable user	543	62.79	1.89	628	54.70	1.81	1	--	--	1	--	--
Tank user	285	32.40	1.70	529	42.89	1.74	**1.49**	1.21 *	1.84 *	1.47 *	1.17 *	1.85 *
Use ENDS Because: they might be less harmful to people around me than cigarettes												
Yes	614	72.31	1.75	1007	85.30	1.06	2.06*	1.57 *	2.69 *	2.10 *	1.59 *	2.76 *
No	245	27.15	1.73	179	14.55	1.06	1	--	--	1	--	--
Use/used e-cigarettes because: They don’t smell												
Yes	538	63.85	1.74	901	76.95	1.48	1.90*	1.49 *	2.41 *	1.86 *	1.43 *	2.41 *
No	322	35.84	1.73	285	22.85	1.49	1	--	--	1	--	--
Use/used e-cigarettes because: It comes/came in flavors I like/liked												
Yes	520	58.11	1.79	905	74.75	1.43	1.99 *	1.58 *	2.49 *	2.04 *	1.62 *	2.55 *
No	337	41.24	1.79	282	25.12	1.44	1	--	--	1	--	--
Use/used e-cigarettes because: Using them helps people to quit smoking cigarettes												
Yes	546	64.57	2.03	877	73.74	1.44	1.51 *	1.20 *	1.90 *	1.52 *	1.20 *	1.92 *
No	315	35.26	2.02	309	26.03	1.44	1	--	--	1	--	--
Use/used e-cigarettes as an alternative to quitting tobacco altogether												
Yes	396	47.53	1.82	736	62.50	1.49	1.70 *	1.37 *	2.11 *	1.71 *	1.37 *	2.13 *
No	464	52.14	1.82	448	37.18	1.48	1	--	--	1	--	--
Use/used e-cigarettes as a way of cutting down on cigarette smoking												
Yes	485	59.07	1.71	931	78.22	1.45	2.40 *	1.90 *	3.02 *	2.38 *	1.86 *	3.05 *
No	376	40.89	1.71	257	21.70	1.46	1	--	--	1	--	--
***Cessation and Dependence***												
Count of times over past 12 months, stopped using tobacco for one day or longer because trying to quit												
0 times	558	64.29	1.62	706	59.26	1.66	1	--	--	1	--	--
1 time	71	8.80	1.13	104	8.88	1.02	0.95	0.64	1.41	0.98	0.66	1.45
2–3 times	153	17.72	1.41	237	20.04	1.28	1.20	0.93	1.55	1.23	0.95	1.61
4+ times	80	9.19	0.98	142	11.82	0.99	1.46 *	1.07 *	2.00 *	1.54 *	1.12 *	2.13 *
Minutes after waking to first cigarette												
less than 5 min	192	23.32	1.67	273	22.75	1.17	1	--	--	1	--	--
5–30 min	303	33.32	1.61	443	36.97	1.61	1.15	0.91	1.46	1.13	0.88	1.46
31–60 min	165	19.71	1.48	220	18.94	1.30	1.00	0.72	1.39	1.00	0.71	1.40
61+ min	195	22.82	1.78	248	20.85	1.43	1.00	0.76	1.32	0.99	0.74	1.33
Minutes after waking to first puff on ENDS												
Missing	552	63.02	1.88	414	35.44	1.50						
Less than 5 min	71	8.41	1.28	138	11.26	0.92	1	--	--	1	--	--
5–30 min	74	8.52	1.00	196	16.73	1.34	1.39	0.86	2.23	1.47	0.82	2.63
31–60 min	53	5.55	0.65	148	12.45	0.95	1.83 *	1.16 *	2.88 *	2.03 *	1.17 *	3.54 *
61+ min	112	14.50	1.42	293	24.12	1.14	1.25	0.80	1.94	1.37	0.79	2.36
Frequency of Daily Cigarette/ENDS Use												
Nondaily (Both Products)	169	19.94	1.39	184	15.58	1.12	1	--	--	1	--	--
Nondaily Cigs, Daily ENDS	40	4.97	0.90	158	13.14	0.98	3.00 *	1.92 *	4.67 *	1.28	0.72	2.26
Daily (Both Products)	41	5.49	0.93	142	12.39	0.98	2.67 *	1.66 *	4.28 *	1.60	0.81	3.14
Daily Cigs, Nondaily ENDS	612	69.60	1.71	705	58.89	1.72	1.02	0.81	1.28	0.63	0.39	1.04
Frequency of Daily ENDS Use, Regardless of Cigarette Use												
Nondaily ENDS	781	89.55	1.21	889	74.47	1.36	1	--	--	1	--	--
Daily ENDS	81	10.45	1.21	300	25.53	1.36	2.79 *	2.05 *	3.81 *	2.04 *	1.39 *	3.01 *
Frequency of Daily Cigarette Use												
Nondaily Cigarette User	209	24.91	1.63	342	28.72	1.44	1	--	--	1	--	--
Daily Cigarette User	653	75.09	1.63	847	71.28	1.44	0.79	0.64	0.97	0.65	0.43	1.00

^a^ Crude-odds ratios correspond to model output using only the given variable as a predictor for past-30 day self-reported ENDS use. ^b^ Adjusted-odds ratios correspond to model output using the given variable as a predictor for past-30 day self-reported ENDS use, including age, gender, race, education, and minutes to first cigarette as model covariates. Corresponds to reference category. * Corresponds to significant odds ratio (*p* < 0.05)
